# Acidic preconditioning of endothelial colony-forming cells (ECFC) promote vasculogenesis under proinflammatory and high glucose conditions in vitro and in vivo

**DOI:** 10.1186/s13287-018-0872-7

**Published:** 2018-05-02

**Authors:** Hebe Agustina Mena, Paula Romina Zubiry, Blandine Dizier, Mirta Schattner, Catherine Boisson-Vidal, Soledad Negrotto

**Affiliations:** 1 Experimental Thrombosis Laboratory, Institute of Experimental Medicine (IMEX), National Academy of Medicine-CONICET, Pacheco de Melo, 3081 Buenos Aires, Argentina; 2 Université Paris Descartes, Sorbonne Paris Cité, Paris, France; 3 INSERM, UMR-S1140, Paris, France

**Keywords:** Type 2 diabetes, Inflammation, Human endothelial colony forming cells, Hind limb ischemia, Acidic preconditioning, Angiogenesis, Vasculogenesis

## Abstract

**Background:**

We have previously demonstrated that acidic preconditioning of human endothelial colony-forming cells (ECFC) increased proliferation, migration, and tubulogenesis in vitro, and increased their regenerative potential in a murine model of hind limb ischemia without baseline disease. We now analyze whether this strategy is also effective under adverse conditions for vasculogenesis, such as the presence of ischemia-related toxic molecules or diabetes, one of the main target diseases for cell therapy due to their well-known healing impairments.

**Methods:**

Cord blood-derived CD34^+^ cells were seeded in endothelial growth culture medium (EGM2) and ECFC colonies were obtained after 14–21 days. ECFC were exposed at pH 6.6 (preconditioned) or pH 7.4 (nonpreconditioned) for 6 h, and then pH was restored at 7.4. A model of type 2 diabetes induced by a high-fat and high-sucrose diet was developed in *nude* mice and hind limb ischemia was induced in these animals by femoral artery ligation. A *P* value < 0.05 was considered statistically significant (by one-way analysis of variance).

**Results:**

We found that acidic preconditioning increased ECFC adhesion and the release of pro-angiogenic molecules, and protected ECFC from the cytotoxic effects of monosodium urate crystals, histones, and tumor necrosis factor (TNF)α, which induced necrosis, pyroptosis, and apoptosis, respectively. Noncytotoxic concentrations of high glucose, TNFα, or their combination reduced ECFC proliferation, stromal cell-derived factor (SDF)1-driven migration, and tubule formation on a basement membrane matrix, whereas almost no inhibition was observed in preconditioned ECFC. In type 2 diabetic mice, intravenous administration of preconditioned ECFC significantly induced blood flow recovery at the ischemic limb as measured by Doppler, compared with the phosphate-buffered saline (PBS) and nonpreconditioned ECFC groups. Moreover, the histologic analysis of gastrocnemius muscles showed an increased vascular density and reduced signs of inflammation in the animals receiving preconditioned ECFC.

**Conclusions:**

Acidic preconditioning improved ECFC survival and angiogenic activity in the presence of proinflammatory and damage signals present in the ischemic milieu, even under high glucose conditions, and increased their therapeutic potential for postischemia tissue regeneration in a murine model of type 2 diabetes. Collectively, our data suggest that acidic preconditioning of ECFC is a simple and inexpensive strategy to improve the effectiveness of cell transplantation in diabetes, where tissue repair is highly compromised.

**Electronic supplementary material:**

The online version of this article (10.1186/s13287-018-0872-7) contains supplementary material, which is available to authorized users.

## Background

Blood vessel endothelium is a key organ involved not only in the regulation of thrombus formation but also in tissue regeneration. Effective endothelial repair and development of new vessels requires the contribution of both angiogenesis, mediated by mature endothelial cells in nearby tissues, and vasculogenesis, supported by bone marrow-derived cells that have the potential to differentiate into mature endothelial cells and are collectively referred to as endothelial progenitor cells [1]. Different cell populations have been reported to play roles in vasculogenesis, but only one population commonly known as endothelial colony-forming cells (ECFC; also known as late outgrowth endothelial progenitor cells) represents an endothelial cell type with potent intrinsic angiogenic capacity and the capability of contributing to vascular repair and de novo blood vessel formation [2, 3]. Vasculogenesis involves ECFC migration from the bone marrow in response to a chemokine gradient including stromal cell-derived factor (SDF)1 and adhesion to the endothelium and/or the extracellular matrix proteins. After binding to the extracellular matrix, integrins initiate signal transduction by recruiting various signaling and adaptor proteins via their cytoplasmic domains such as focal adhesion kinase (FAK), which plays a key role in integrin signaling and cell migration and tissue invasion [4]. Finally, ECFC proliferate and differentiate into mature endothelial cells and are organized into three-dimensional structures that, together with support cells, form the new functional vessel.

The formation of new blood vessels in adults usually occurs in the context of an ischemic and/or inflammatory microenvironment which may disturb not only transplanted ECFC but also endogenous and local progenitors due to the presence of high level of stressful signals as proinflammatory cytokines, such as tumor necrosis factor (TNF)α, interleukin (IL)-6, -1, and -18 [5], and damage-associated molecular patterns (DAMP), such as extracellular histones, monosodium urate (MSU) crystals, and others. The ischemic milieu may become even more destructive in the presence of cardiovascular risk factors. For instance, in type 2 diabetes, a condition associated with high glucose, obesity, and/or cholesterol, critical limb ischemia and impaired wound healing are frequent complications. Moreover, low levels and impaired functionality of circulating endothelial progenitor cells has been reported in diabetic patients (reviewed in [6]); these are reasons that explain why they are considered a target population for regenerative cell therapy.

We have previously demonstrated that acidic preconditioning of ECFC exacerbates their angiogenic responses including proliferation, SDF1-driven chemotaxis, and capillary-like tubule formation, as well as revascularization of the postischemic hind limb in mice without baseline disease [7]. We here analyze whether this strategy is also effective under adverse conditions for vasculogenesis, such as the presence of ischemia-related toxic molecules or in diabetes. We demonstrate that acidic preconditioning promoted ECFC adhesion and the release of pro-angiogenic molecules. In addition, this strategy improved ECFC survival and angiogenic activity in the presence of proinflammatory and damage signals present in the ischemic milieu, even under high glucose conditions, and improved their therapeutic potential for postischemia tissue regeneration in a murine model of type 2 diabetes.

## Methods

### Endothelial cell cultures

Human umbilical vein endothelial cells (HUVEC) and ECFC were obtained from umbilical cord vein and blood, respectively, and characterized as previously described [7, 8]. Both ECFC and HUVEC were expanded and maintained in endothelial growth medium 2 (EGM2; Lonza, Walkersville, MD, USA).

### Experimental design

The culture medium was acidified by the addition of a precalculated volume of isotonic HCl (1 N). Acid preconditioning was achieved by incubating ECFC at pH 6.6 for 6 h and then the medium was replaced by fresh EGM2 adjusted to physiologic pH (7.4) at 37 °C in a humidified atmosphere with 5% CO_2_ [7]. The pH of the medium was measured at 37 °C and averaged 7.40 ± 0.05 and 6.63 ± 0.04; pH levels remained steady for the first 8 h [7]. ECFC were collected immediately after preconditioning for in vitro and in vivo experiments. MSU crystals (InvivoGen, San Diego, CA, USA), human recombinant histones (New England Biolabs, Ipswich, MA, USA), d-(+)-glucose (Sigma, St. Louis, MO, USA) and/or TNFα (Cell Signaling Technology, Danvers, MA, USA) were added after preconditioning. The pan-caspase inhibitor Z-VAD-fmk (carbobenzoxy-valyl-alanyl-aspartyl-[O-methyl]-fluoromethylketone; Biomol, Plymouth Meeting, PA, USA) was added 15 min before proapoptotic stimuli.

### ECFC adhesion under static conditions

ECFC (2 × 10^4^) were stained with the fluorescent dye carboxyfluorescein succinimidyl ester (CFSE; eBioscience, San Diego, CA, USA) and then seeded in 48-well culture dishes previously coated with recombinant bovine fibronectin (10 μg/mL; Sigma), rat tail type-I collagen (10 μg/mL; BD Biosciences) or TNFα-activated HUVEC. After 30 min or 2 h, plates were washed and the number of adherent cells in five random fields was counted using ImageJ software [9].

### ECFC adhesion under dynamic conditions

ECFC were stained with CFSE (eBioscience) and perfused onto a precoated chamber slide containing rat tail type-I collagen (10 μg/mL) or TNFα-activated HUVEC. Perfusion of ECFC was achieved using a flow chamber (GlycoTech, Gaithersburg, MD, USA) at 0.6 mL/H (shear force, 1 dyne/cm^2^) for 15 min. Cells were fixed with paraformaldehyde (1%) for 15 min and stained adherent cells were examined by phase-contrast confocal fluorescence microscopy using a FV-1000 microscope (Olympus, Tokyo, Japan). The number of adherent cells was determined by cell count using Image J [9].

### Flow cytometry studies

Cells were stained with antibodies against β1 (Santa Cruz Biotechnology, Dallas, TX, USA), αVβ3, α5 (R&D Systems, Inc., Minneapolis, MN, USA), ICAM, or E-selectin (BD Bioscience) for 30 min conjugated with different fluorochromes. For caspase-3 activation, cells were fixed, permeabilized, and then stained with fluorescein isothiocyanate (FITC)-conjugated monoclonal antibody against the active fragment of caspase-3 (BD Biosciences) for 30 min. For reactive oxygen species (ROS) generation, ECFC were incubated with dihydrorhodamine (5 mM) for 60 min at 37 °C. Sample acquisition and analysis were performed by flow cytometry (FACScalibur, BD Biosciences, San Jose, CA, USA) using FCS Express V3 (De Novo Software, Glendale, CA, USA).

### Western blotting

ECFC were placed onto fibronectin for 1 h and FAK phosphorylation levels were determined by Western blot. In brief, cell lysates were electrophoresed and transferred to a nitrocellulose membrane. After blocking, membranes were incubated first with an antibody against human FAK (phospho Y397; Abcam, Cambridge, UK) and then with horseradish peroxidase (HRP)-conjugated secondary antibody. Each membrane was reprobed with an antibody against actin to calculate the relative integrated optical density (IOD) using Gel-Pro analyzer 3.1 software.

### Quantification of pro-angiogenic factor levels

The concentration of basic fibroblast growth factor (bFGF), IL-10 (Biolegend, San Diego, CA, USA), transforming growth factor (TGF)β1, IL-8, IL-4 (eBioscience), vascular endothelial growth factor (VEGF; R&D Systems, Inc., Minneapolis, MN, USA), platelet-derived growth factor (PDGF; Abcam), and epidermal growth factor (EGF; Life Technologies, Carlsbad, CA, USA) in ECFC culture supernatants were measured by enzyme-linked immunosorbent assay (ELISA) at the indicated time points following the manufacturer’s instructions.

### Measurement of cell death

Nuclear morphology was analyzed by fluorescence microscopy after double staining of ECFC with ethidium bromide and acridine orange (Sigma). Phosphatidylserine expression was assessed after cell staining with Annexin V (BD) and propidium iodide (PI) and analyzed by flow cytometry [8].

### IL-1β measurement

Cells were stimulated with MSU for 24 h, and Golgi stop (BD Bioscience; 0.65 μl/ml) was added 4 h before fixation and permeabilization with Cytofix-Cytoperm (BD Bioscience). Cells were then stained with anti-human IL-1β monoclonal antibody conjugated with FITC (Biolegend) and the percentage of positive cells was analyzed by flow cytometry.

### Measurement of cell proliferation

ECFC (1 × 10^4^) were seeded on 96-well plates and proliferation was assessed by cell counting in a Neubauer chamber after 48 h. Counts were performed in triplicate by one analyst under a 40× objective.

### Scratch assay

Confluent ECFC monolayers were scratched by scraping with a pipette tip and the extent of cell migration into the scratched area was photographed under the microscope at different time points. The gap surface area was measured by analyzing the images with ImageJ software and the percentage of gap closure was calculated as [(gap area at 0 h – gap area at *x* h)/gap area at 0 h] ×100 [7].

### Chemotaxis assay

Chemotaxis was examined in 24-well micro-chemotaxis chambers with 8-μm pore size (Sigma). ECFC (1.5 × 10^4^) resuspended in endothelial basic medium 2 (Lonza) supplemented with 5% fetal calf serum (FCS; Gibco, Grand Island, NY, USA) were placed in the upper chamber and allowed to migrate toward the lower chamber containing the same medium with SDF1 (20 ng/mL; Abcys, Paris, France). Migration was allowed to proceed for 6 h at 37 °C. Cells remaining on the upper surface of the filters were mechanically removed and then membranes were fixed with 1% formaldehyde and stained with Giemsa. The number of migrated cells was determined by counting under a high-power microscope (Olympus) [8].

### In vitro tubule formation assay

ECFC (1.5 × 10^4^) were seeded on 96-well plates coated with reduced growth factor basement membrane matrix (Geltrex™; Gibco). After 18 h, tubule formation was examined by phase-contrast microscopy and the total number of branch points was quantified on the entire surface of each well using ImageJ [7].

### Animal studies

Eight-week-old C57BL/6 J male athymic *nude* mice housed in a controlled environment were randomly divided into two groups: 1) standard chow and water for rodents (normoglycemic group); and 2) a 60% kcal high-fat diet and 10% sucrose-supplemented water (diabetic group). The high-fat chow contained 15% protein, 36% fat (butter), 37% carbohydrate (18% as sucrose), minerals (4.5%), and starch (14.5%) (SAFE AUGY, U8978P Version 0019). Plasma glucose levels were monitored weekly in blood from the tail plexus using a glucometer (OneTouch Verio®IQ, LifeScan, France). After 10 weeks, mice underwent surgery to induce unilateral hind limb ischemia by ligation of the right femoral artery as previously described [7]. Nonpreconditioned or preconditioned ECFC (1 × 10^5^ in phosphate-buffered saline (PBS)) or PBS alone (vehicle) were administered 5 h after occlusion in the retro-orbital plexus, and the ischemic/normal limb blood flow ratio was determined on days 7 and 14 using the laser Doppler perfusion imaging system PeriScan Pim3 (Perimed, Crappone, France). Gastrocnemius muscles were surgically removed for histological analysis. Vascular density was determined by counting the number of blood vessels in entire Masson’s trichrome sections and were expressed as vessels per mm^2^. Inflammation score (arbitrary units) was determined by analyzing the presence of vacuoles, centered and hypertrophic nuclei, and leukocyte infiltrate in hematoxylin and eosin (H&E) sections.

### Statistical analysis

Results are expressed as means ± SEM. Significant differences (*P* < 0.05) were identified by one-way analysis of variance (ANOVA) followed by the Bonferroni test using the GraphPad software package (PRISM Version 5.0, San Diego, CA, USA).

## Results

### Preconditioned ECFC exhibit enhanced adhesive properties and release of pro-angiogenic cytokines

**Fig. 1:**
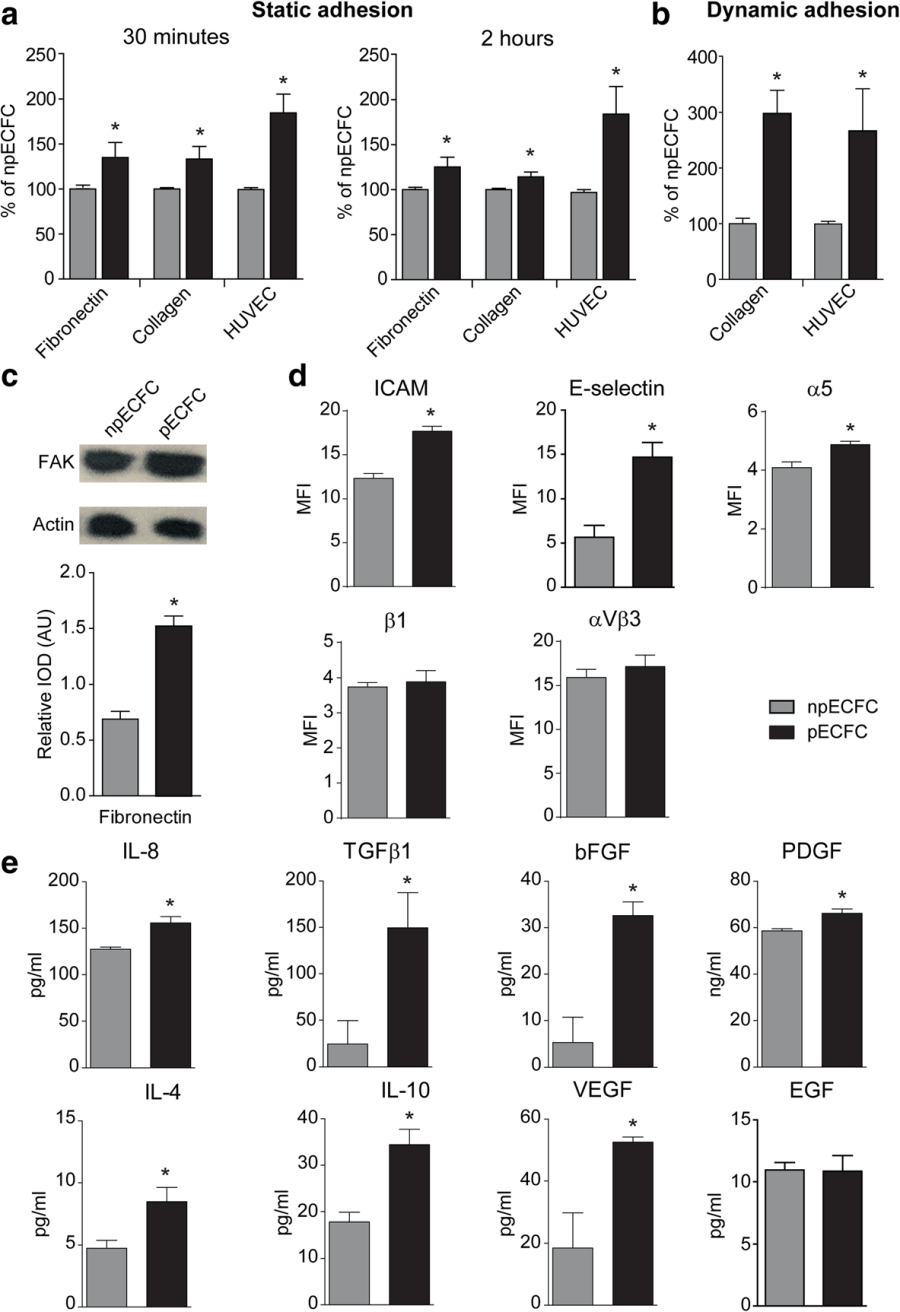
Acidic preconditioning increased endothelial colony-forming cell (EFCF) adhesion and pro-angiogenic factor release. **a** Nonpreconditioned (npECFC) or preconditioned ECFC (pECFC) (grey and black bars, respectively) were seeded onto fibronectin, collagen type I, or TNFα-activated human umbilical vein endothelial cells (HUVEC) and, after 30 min or 2 h, the number of adherent cells was counted (*n* = 5). **b** ECFC were perfused onto collagen or TNFα-activated HUVEC for 15 min at shear force 1 dyn/cm^2^ and the number of adherent cells was counted (*n* = 4). **c** ECFC were seeded onto fibronectin for 30 min and focal adhesion kinase (FAK) phosphorylation was determined by Western blot. Each membrane was reprobed with anti-actin antibody to calculate the relative integrated optical density (IOD) (*n* = 4). **d** ECFC were stained with antibodies against ICAM, E-selectin, α5, β1, or αVβ3 and then analyzed by flow cytometry (*n* = 3). **e** Interleukin (IL)-8, transforming growth factor (TGF)β1, basic fibroblast growth factor (bFGF), platelet-derived growth factor (PDGF), and IL-4 or IL-10, vascular endothelial growth factor (VEGF) and epidermal growth factor (EGF) cytokines were measured in the supernatants of npECFC or pECFC after 24 or 48 h, respectively (*n* = 5). *p < 0.05 vs npECFC. AU, arbitrary units; MFI, mean fluorescence intensity

We have previously demonstrated that acidic preconditioning increased ECFC proliferation, migration, and tubule formation [7]. We now extend those findings and demonstrate that this strategy also promotes other ECFC angiogenic responses. Preconditioned ECFC exhibited both enhanced nascent (30 min) and focal (2 h) adhesions to fibronectin, type-I collagen, and TNFα-activated HUVEC either under static conditions (Fig. 1a) or physiological shear stress conditions (Fig. 1b). These enhanced adhesive abilities were associated with higher FAK phosphorylation levels on Tyr576 triggered by fibronectin (Fig. 1c), and a slight but significant increase in integrin α5, ICAM, and E-selectin, but not β1 or αVβ3 (Fig. 1d).

In addition, we found that acidic preconditioning enhanced the release of several pro-angiogenic and anti-inflammatory molecules. While higher levels of TGFβ1, bFGF, PDGF, IL-8, IL-4, VEGF, and IL-10 were detected in supernatants from preconditioned ECFC, EGF showed no significant differences between treatments (Fig. 1e).

In agreement with our previous study [7], the formation of capillary-like structures was highly increased (64 ± 9%) when ECFC were placed on Geltrex™ matrix immediately after preconditioning. Interestingly, we now observed that the angiogenic advantage of preconditioned ECFC remains intact after 8 h post-preconditioning (exhibiting an increase of 59 ± 7%), then decay after 18 h (39 ± 3%) and 24 h (21 ± 4%), and finally is completely lost after 48 h (1 ± 5%).

### Acidic preconditioning protects ECFC from death induced by different cytotoxic stimuli

**Fig. 2:**
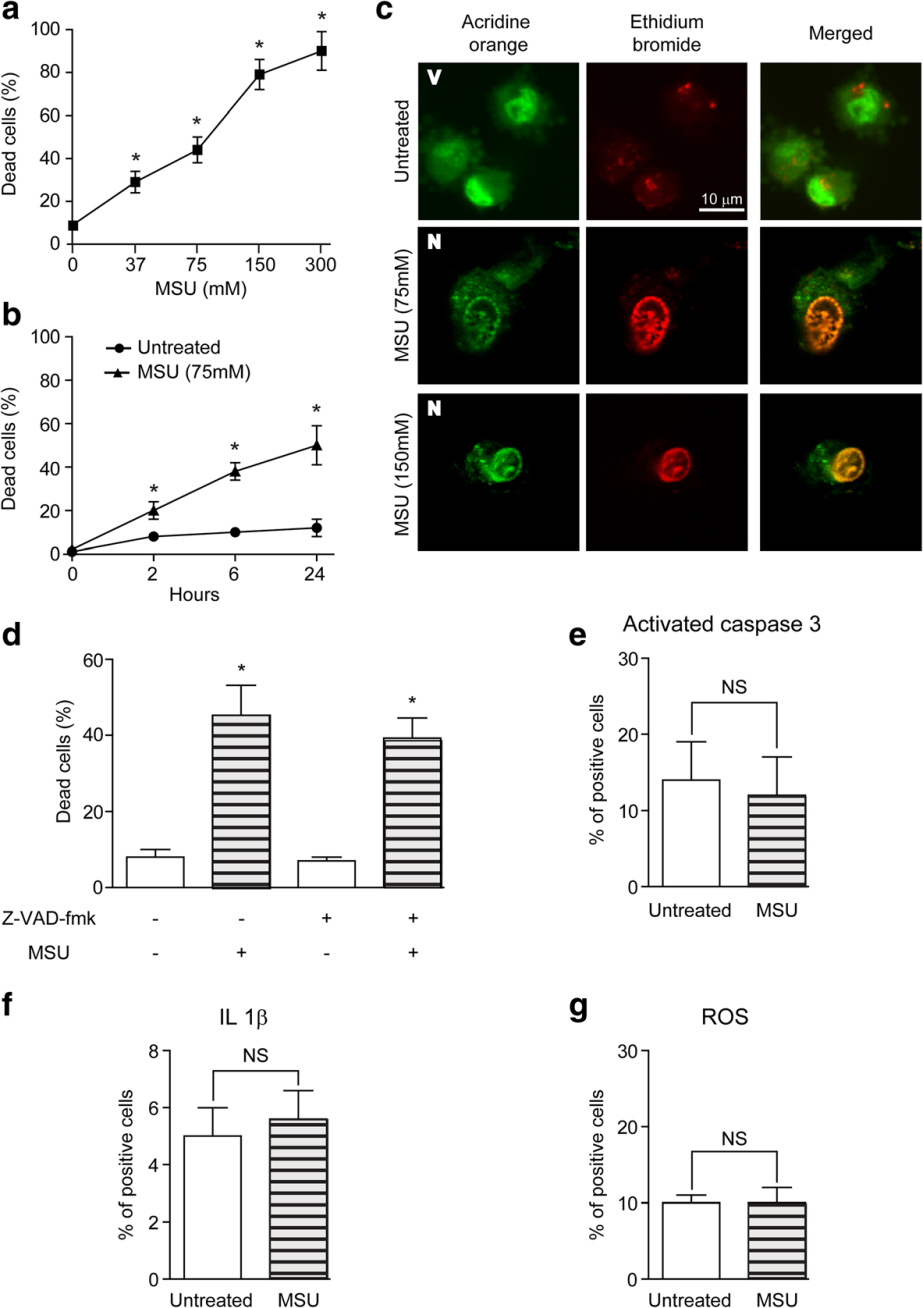
Monosodium urate (MSU) induced endothelial colony-forming cell (EFCF) necrosis. **a** ECFC were treated with MSU at the indicated concentration and the cell death was analyzed by fluoresce microscopy after 24 h (*n* = 5). **b** Kinetics of ECFC death were studied by fluorescence microscopy (*n* = 4). **c** Representative images of viable (V) and necrotic (N) cells are shown. Original magnification, 1200×. Scale bar = 10 μm. **d** The percentage of cell death was measured by fluorescence microscopy in the presence or absence of Z-VAD-fmk (30 μM). The percentage of positive cells for active caspase-3 (**e**), interleukin (IL)-1β levels (**f**), and reactive oxygen species (ROS) formation (**g**) were analyzed by flow cytometry after 24 h (*n* = 3). *p < 0.05 vs untreated. NS, not significant

Endothelial progenitor cells that migrate to injured tissues encounter a hostile milieu of stress signals and DAMP that might undermine its survival and functionality. Because MSU crystals are considered one of the most abundant DAMP in ischemic and necrotic tissues [10, 11], we first analyzed their effect on ECFC and found that MSU crystals triggered ECFC death in a concentration- and time-dependent manner (Fig. 2a, b). Nuclear morphology changes and the observation that cellular death was independent of caspases, NLRP3 inflammasome activation, or ROS formation indicated that MSU induced necrosis of ECFC (Fig. 2c–g).

**Fig. 3:**
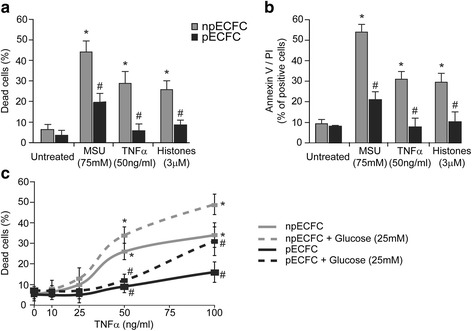
Preconditioned endothelial colony-forming cells (EFCF) are more resistant to DAMP- or TNFα-induced cell death. Nonpreconditioned or preconditioned ECFC (npECFC or pECFC, respectively) were incubated with monosodium urate (MSU), tumor necrosis factor (TNF)α, or histones at the indicated concentrations. **a** The nuclear morphology was analyzed by fluorescence microscopy and (**b**) the percentage of annexin V and propidium iodide (PI) double-positive cells was measured by flow cytometry after 24 h (*n* = 6). **c** ECFC were incubated with high glucose, TNFα, or their combination and cell death was analyzed after 24 h (*n* = 6). *p < 0.05 vs untreated npECFC; ^#^*p* < 0.05 vs npECFC with the same treatment

The fact that stress signals that are highly present in the inflammatory and ischemic microenvironment can trigger different programs of cellular death prompted us to elucidate whether acidic preconditioning protects ECFC from three different death stimuli: MSU (necrosis), TNFα (apoptosis), and histones (pyroptosis). Interestingly, MSU-, TNFα-, and histone-mediated cytotoxicity were significantly reduced in preconditioned ECFC when compared with nonpreconditioned ECFC (Fig. 3a). Similar results were observed by flow cytometry after annexin V and PI staining (Fig. 3b).

Although diabetes, especially the type 2 class, is one of the main target diseases for cell therapy due to its well-known healing impairments, studies regarding the effect of high glucose conditions on ECFC survival are scarce and controversial [12]. Our results revealed that high glucose alone failed to induce ECFC death (Fig. 3c). However, TNFα-induced apoptosis of ECFC was significantly increased under high glucose conditions and these effects were observed in both untreated and preconditioned ECFC, although cytotoxicity was lower in the latter (Fig. 3c). MSU- or histone-induced toxicity was not significantly affected by glucose (Additional file 1). Mannitol (as an osmotic control) and normal glucose concentration (5 mM) were run in parallel and, in both cases, results were no different from untreated cells (data not shown).

### ECFC angiogenic activity is enhanced by acidic preconditioning under proinflammatory and high glucose conditions in vitro

We next analyzed whether acid preconditioning, in addition to its cytoprotective effect, improved the angiogenic responses of ECFC exposed to inflammatory but nontoxic concentrations of TNFα in the presence or absence of high glucose. ECFC adhesion was not significantly affected by high glucose and/or TNFα, except for the improved adhesion of preconditioned ECFC to activated HUVEC which was significantly reduced by high glucose or TNFα and completely abrogated by their combination (Additional file 2).

**Fig. 4:**
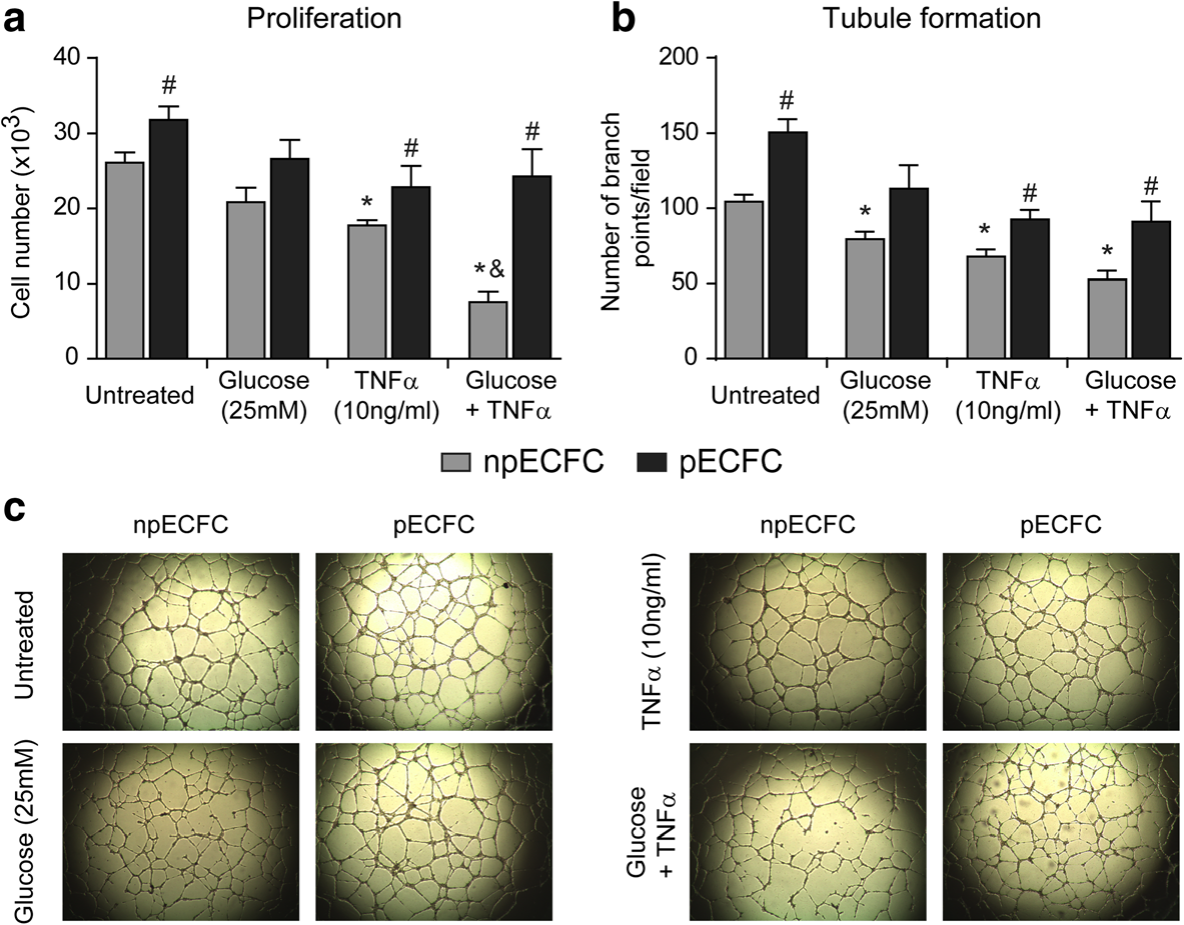
Acidic preconditioning improved endothelial colony-forming cell (EFCF) proliferation and tubule formation under adverse conditions. Nonpreconditioned or preconditioned ECFC (npECFC or pECFC, respectively) were treated with high glucose, tumor necrosis factor (TNF)α, or their combination, and the different angiogenic responses were measured. **a** Cell counts were performed after 48 h using a Neubauer chamber (*n* = 4). **b** Cells were seeded on Geltrex™ matrix and the number of branch points per field was measured after 18 h. **c** Representative images of six different experiments. Original magnification, 40×. *p < 0.05 vs untreated npECFC; ^#^*p* < 0.05 vs npECFC with the same treatment; ^&^*p* < 0.05 vs TNFα or glucose alone

**Fig. 5:**
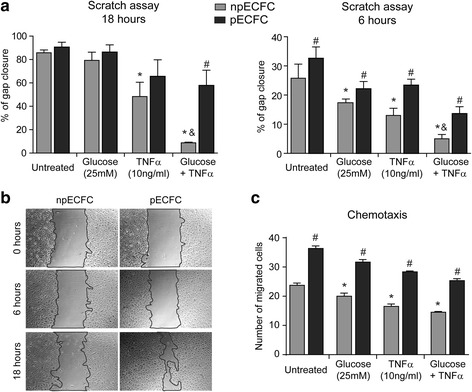
Acidic preconditioning improves endothelial colony-forming cell (EFCF) migration under stressful conditions. Nonpreconditioned or preconditioned ECFC (npECFC or pECFC, respectively) were treated with high glucose, tumor necrosis factor (TNF)α, or their combination, and the different angiogenic responses were measured. **a** The extent of cell migration into the scratched area was measured after 0, 6, and 18 h (*n* = 5). **b** Images show the kinetic of gap closure in npECFC or pECFC treated with both high glucose and TNFα. Original magnification, 40×. **c** Chemotaxis in response to SDF1 (20 ng/ml) was determined using transwell inserts. The number of migrated cells was counted after 6 h (*n* = 4). *p < 0.05 vs untreated npECFC; ^#^*p* < 0.05 vs npECFC with the same treatment; ^&^*p* < 0.05 vs TNFα or glucose alone

Regarding ECFC proliferation, TNFα alone induced a significant antiproliferative effect in nonpreconditioned cells, which was more pronounced when combined with high glucose, whereas almost no inhibition was observed in preconditioned ECFC (Fig. 4a). Similar results were observed when tubule formation on Geltrex™ matrix was assessed (Fig. 4b, c). No proliferation or tubule formation was observed when ECFC were cultured in the cytokine-free medium EBM2 (negative control). We also found that the extent of proliferation and migration into the cell monolayer scratched area after 18 h was significantly impaired by TNFα alone or combined with high glucose in untreated ECFC, while these effects were significantly prevented by acidic preconditioning (Fig. 5a, b). A similar gap closure inhibition was detected after 6 h when proliferation barely contributes to this process, revealing a direct suppressive effect on cell migration mediated by TNFα and high glucose, which was again significantly lower in preconditioned ECFC (Fig. 5a, b). These results were confirmed by analyzing ECFC migration in response to SDF1 using transwell inserts (Fig. 5c).

### Postischemia tissue regeneration is improved by preconditioned ECFC in normoglycemic and type 2 diabetic mice

**Fig. 6:**
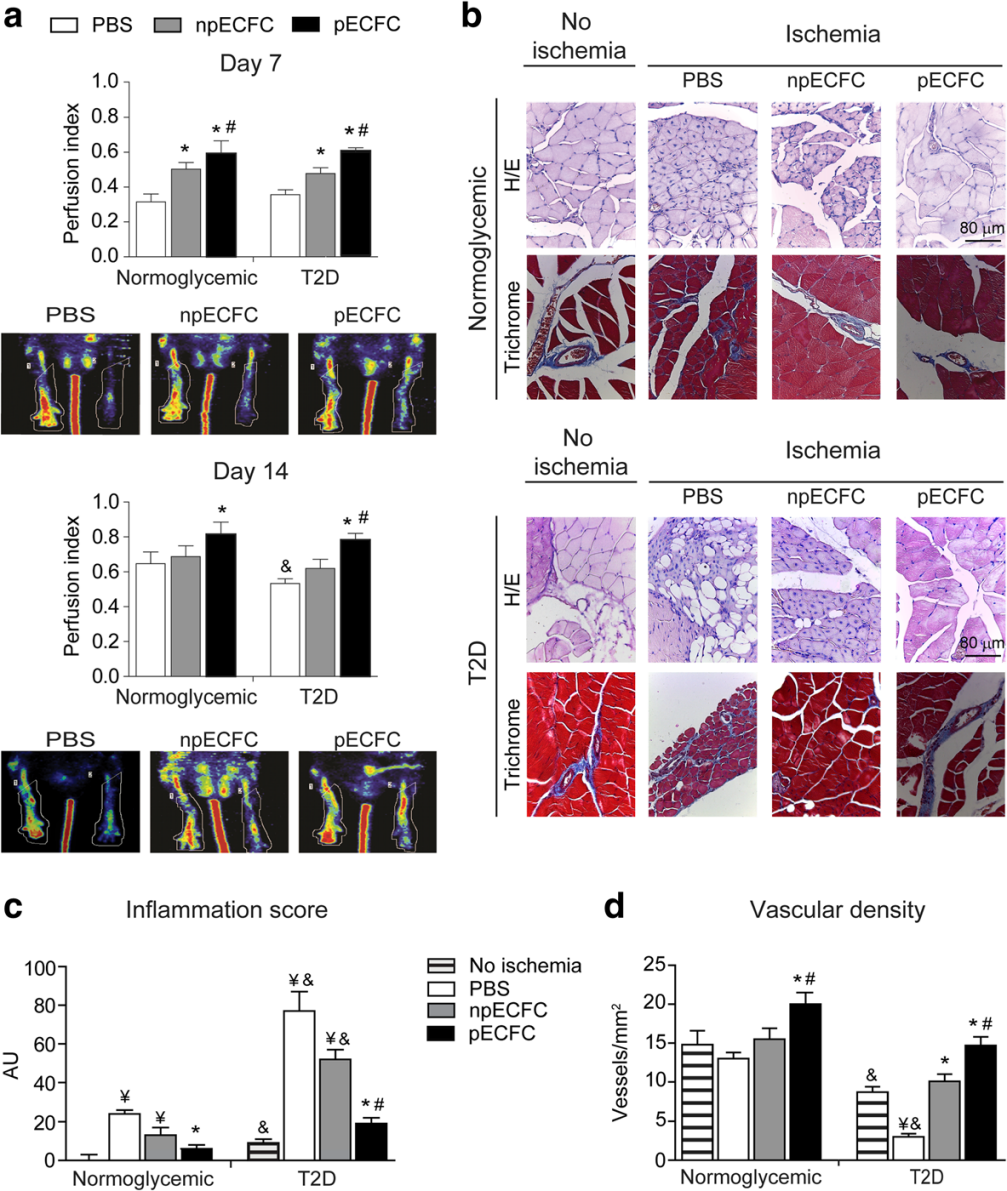
Acidic preconditioning enhanced endothelial colony-forming cell (EFCF)-mediated neovascularization in ischemic hind limbs of normoglycemic and diabetic mice. Phosphate-buffered saline (PBS), and nonpreconditioned or preconditioned ECFC (npECFC or pECFC, respectively) were infused intravenously in normoglycemic and type 2 diabetic (T2D) mice 5 h after ischemia-inducing surgery. **a** Perfusion index was calculated by Doppler analysis after 7 and 14 days postischemia. Representative images of T2D animals are shown. **b** Histologic analysis of gastrocnemius muscles, stained with hematoxylin and eosin (H/E) or Masson’s trichrome, was performed after 14 days postischemia. Original magnification, 400×. Scale bar = 80 μm **c** Inflammation score was calculated in H/E stained sections. **d** Vascular density was measured in Masson’s trichrome stained sections. *n* = 6 per group. *p < 0.05 vs PBS; ^#^*p* < 0.05 vs npECFC; ^&^*p* < 0.05 vs the same treatment in the normoglycemic group; ^¥^*p* < 0.05 vs no ischemia. AU, arbitrary units

We have previously demonstrated that after hind limb ischemia induction in normoglycemic mice blood flow recovery was significantly increased by the administration of preconditioned ECFC (versus nonpreconditioned ECFC- and PBS-treated groups) as measured by Doppler scanning [7]. We now confirm and extend our previous findings, performing a deeper analysis of the muscle histology. In agreement with our previous study, Doppler analysis showed that a single intravenous injection of preconditioned ECFC increased the ischemic to nonischemic leg blood flow ratio relative to nonpreconditioned ECFC- or PBS-treated mice (Fig. 6a). Moreover, histologic analysis of the gastrocnemius muscles at high and low magnifications (Fig. 6b and Additional file 3, respectively) revealed that the injection of nonpreconditioned ECFC reduced local inflammation (Fig. 6c) and had no effect on vascular density (Fig. 6d) when compared with PBS-treated animals. Interestingly, both parameters were significantly improved after administration of preconditioned ECFC.

Type 2 diabetic patients are a target population for cell therapy since critical limb ischemia and impaired wound healing are frequent complications. According to the severity of the disease, these patients might develop diabetic foot syndrome, a clinical entity that might result in infection and amputation [13]. Having shown in vitro that acidic preconditioning improves the angiogenesis activity of ECFC even under inflammatory or hyperglycemic conditions, we next aimed to elucidate whether preconditioning is an effective strategy to improve postischemia tissue regeneration in a murine model of type 2 diabetes. Nude mice at the age of 8 weeks were fed either chow or diabetogenic diet for a total of 10 weeks, when hyperglycemia was established (Table 1). Despite the similar total body fat in both groups (Table 1), the amount of epididymal, inguinal, and interscapular fat was higher in diabetic mice compared with normoglycemic ones. According to Doppler analysis, the muscle perfusion index in diabetic animals was similar to normoglycemic mice after 7 days, but significantly lower after 14 days (Fig. 6a). Nonpreconditioned ECFC significantly enhanced foot perfusion in the diabetic ischemic limb compared with PBS-treated animals after 7, but not 14, days postischemia, whereas preconditioned ECFC further improved blood flow at both 7 and 14 days compared with the PBS and nonpreconditioned ECFC groups (Fig. 6a). In addition to reducing blood flow, histologic analysis of the gastrocnemius muscles at high and low magnifications (Fig. 6b and Additional file 3, respectively) showed that administration of preconditioned ECFC significantly diminished the inflammation score (Fig. 6c) and increased vascular density (Fig. 6d) compared with the nonpreconditioned ECFC or PBS groups. Of note, there were no episodes of lower limb necrosis or defects in ambulation in any group.

**Table 1:** Mice glycemia and weight evolution

Weeks of diet	Glycemia (g/L)		Weight (g)	
	Normoglycemic	Type 2 diabetes	Normoglycemic	Type 2 diabetes
1	1.55 ± 0.04	1.43 ± 0.05	29.49 ± 0.28	28.81 ± 0.29
2	1.53 ± 0.05	1.42 ± 0.05	30.19 ± 0.29	29.81 ± 0.34
3	1.30 ± 0.05	1.31 ± 0.05	32.94 ± 0.37	30.30 ± 0.35
4	1.36 ± 0.04	1.11 ± 0.05	32.85 ± 0.39	32.55 ± 0.38
5	1.45 ± 0.07	1.74 ± 0.07*	33.49 ± 0.55	33.67 ± 0.40
6	1.24 ± 0.05	1.85 ± 0.06*	34.36 ± 0.57	36.00 ± 0.62
7	1.42 ± 0.09	1.93 ± 0.05*	35.93 ± 0.88	36.41 ± 0.63
8	1.52 ± 0.08	2.20 ± 0.05*	35.54 ± 0.60	36.01 ± 0.53
9	1.47 ± 0.05	2.03 ± 0.06*	36.23 ± 0.75	35.57 ± 0.62
10 (15 min postsurgery)	1.53 ± 0.07	2.40 ± 0.08*	35.45 ± 0.87	35.58 ± 0.90
11 (7 days postsurgery)	1.54 ± 0.03	2.24 ± 0.05*	33.51 ± 0.60	35.55 ± 0.60
12 (14 days postsurgery)	1.26 ± 0.06	2.13 ± 0.07*	35.13 ± 0.58	35.70 ± 0.62

*p < 0.05 vs normoglycemic by ANOVA

## Discussion

ECFC recruitment to sites of neoangiogenesis involves ECFC migration and adhesion to activated endothelium, as well as a direct interaction with the extracellular matrix—key steps to a successful engraftment. Cell adhesion is initiated by the formation of unstable receptor-matrix complexes (nascent adhesions) that might disassemble and disappear or further mature into stable focal adhesions [4]. Since the regulation of ECFC adhesion by acidic preconditioning was not analyzed in our former study [7], we now performed a deep analysis of this process and provided strong evidence that acidic preconditioning of ECFC enhanced nascent and focal adhesion to fibronectin, collagen, and activated HUVEC under static and physiological shear stress conditions. Interestingly, higher levels of FAK phosphorylation were found in preconditioned ECFC compared with nonpreconditioned ECFC, reinforcing the idea that focal adhesion is augmented by acidosis. FAK is an integrin-dependent tyrosine phosphorylated protein localized at focal adhesion sites that, beyond the classic role in adhesion and migration, also regulates several biological processes including cell motility, differentiation, angiogenesis, and survival via the FAK/Src/Paxillin cascade [4, 14]. Indeed, endothelial integrins might be responsible, at least in part, for the acidic preconditioning-mediated pro-angiogenic effect since a higher expression of the integrin α5, involved in the interaction between ECFC and matrix proteins, was found in preconditioned ECFC. Moreover, ICAM and E-selectin, which mediate their contact with leukocytes and endothelial cells, were also elevated after this procedure.

The release of pro-angiogenic proteins from ECFC was another pro-angiogenic function improved by preconditioning. bFGF, TGFβ1, IL-8, IL-4, VEGF, PDGF, and IL-10 were highly increased in preconditioned ECFC supernatants, while EGF was also secreted with no significant differences between groups. The paracrine action of ECFC has been underestimated when compared with early endothelial progenitor cells, which have a hematopoietic origin and release enormous levels of cytokines and growth factors. Nonetheless, it is currently well known that the ECFC secretome is crucial for the regeneration of damaged tissues [2].

Despite the therapeutic potential of ECFC, clinical studies are still inconclusive regarding the effectiveness of ECFC administration for tissue regeneration. One of the limitations of this practice is the low viability of the transplanted cells in the ischemic and inflammatory microenvironment, probably due to high levels of harmful signals known as DAMP, as well as the presence of cardiovascular risk factors. In fact, we have previously demonstrated that extracellular histones induced mostly pyroptosis of ECFC [8], while TNFα is a well-known proapoptotic cytokine [15, 16]. Under ischemia conditions, intracellular uric acid formation is enhanced [17] and a vast amount of this substance is released after cell death forming MSU crystals, one of the most abundant DAMP in the ischemic microenvironment. We here show that MSU induced ECFC necrosis. A programmed cell death process was disregarded since the MSU toxic effect was independent of caspases, NLRP3 inflammasome activation, and ROS formation. Interestingly, we show that, despite the different cell death program triggered by each stimulus, acidic preconditioning protects ECFC with great efficiency from the harmful effects of MSU, TNFα, and extracellular histones.

The proinflammatory cytokine TNFα is highly augmented in plasma and tissues of patients with several cardiovascular and inflammatory diseases and is considered a risk factor for developing these clinical conditions [18]. Most studies that have investigated the effect of this molecule on endothelial progenitor cells agree that high concentrations of TNFα (> 20 ng/mL) induce apoptosis whereas low concentrations (~ 10 ng/mL) reduce angiogenic responses such as proliferation and tubulogenesis [15, 16, 18]. Following same line of evidence, we here found that TNFα exerted a significant proapoptotic effect at a concentration of 50 ng/mL and, at non-toxic levels (10 ng/mL), inhibited ECFC proliferation, migration, and tubule formation. On the other hand, although diabetes is considered one of the main target diseases for cell therapy due to the frequent ischemic events and wound healing impairments, studies regarding the effect of high glucose conditions on ECFC survival are scarce and controversial. While some studies described a direct cytotoxic effect [19, 20], others are in full agreement with our findings showing that high glucose alone failed to induce apoptosis but inhibited ECFC angiogenic activity [12, 18, 21]. We also found that high glucose potentiated apoptosis triggered by TNFα, an effect that was significantly prevented by acidic preconditioning.

It has been previously shown that TNFα impaired ECFC angiogenic functions through upregulation of p38 MAPK and downregulation of AKT/PI3K [22, 23], and that high glucose inhibited ECFC proliferation and differentiation in a p38 MAPK-dependent manner [19, 20]. Since we have previously demonstrated that acidic preconditioning of ECFC augmented tubulogenesis through p38 MAPK inhibition and increased proliferation through AKT/PI3K activation [7], it is conceivable that these pathways are responsible for acidosis-mediated ECFC protection from TNFα and/or high glucose anti-angiogenic effects.

We showed that acidic preconditioning of late outgrowth endothelial progenitor cells derived from cord and peripheral blood enhances their angiogenic activity in vitro and increased blood flow (Doppler analysis) in a hind limb ischemia model in C57BL/6 J athymic *nude* mice with no baseline disease [7]. In the present study, we further extended those findings by demonstrating that preconditioned ECFC not only augmented blood flow but also increased vascular density and reduced the inflammation score as revealed by histological analysis of gastrocnemius muscle sections. Although endogenous recovery from ischemia in the PBS-treated animals at day 14 was elevated, this is expected for immunocompromised mice in general and this mouse strain in particular [24]. Obesity-related disorders, including diabetes and hypertension, are associated with impaired endothelial progenitor cell functions [25]. In fact, their mobilization in response to injury is impaired in diabetic mice, and glucose-intolerant individuals exhibit lower levels of circulating endothelial progenitor cells [26, 27]. A chronic high-fat high-sucrose (HFHS) diet is a well-established method to promote obesity and insulin resistance, resulting in a type 2 diabetes-like condition (hyperglycemia) in murine models [28, 29]. Although it has been extensively described in the wild-type C57BL/6 J strain, we here described for the first time that this model can be successfully developed in C57BL/6 J athymic *nude* mice. Blood glucose levels in these mice were similar to the wild type after 10–12 weeks of HFHS diet [28, 30]. Regarding the body weight, although a 20–30% increase was reported in the existing models [28, 30], no differences were observed between groups in our *nude* mice after 10 weeks of treatment, but a significant weight gain (24%) in diabetic animals was detected after 18 weeks of diabetogenic diet. Hyperactivity was a frequent observation within the first weeks of a high-fat and high-sugar regimen, which may explain why weight gain was slower than expected; however, an increased amount of adipose tissue was evident in diabetic mice macroscopically (epididymal, inguinal, and interscapular fat accumulation) and microscopically (lipid vacuoles within the muscle fibers). The diabetogenic diet also reduced blood flow recovery after hind limb ischemia, which was significantly different from normoglycemic mice only after 14 days by Doppler scanning. Muscle histology was consistent with Doppler analysis as ischemia induced an exacerbated inflammatory response and lower vascular density compared with nondiabetic animals. Although administration of untreated ECFC ameliorated the impairment of revascularization and tissue inflammation in diabetic animals, these effects were significantly improved after treatment with preconditioned ECFC. Moreover, preconditioned ECFC was the only treatment that significantly restored blood flow in diabetic gastrocnemius muscles at 14 days after ischemia. In addition to its predictable pro-angiogenic activity, our progenitor cells also exhibited anti-inflammatory effects reflected by small numbers of vacuoles, centered and hypertrophic nuclei, and infiltrated leukocyte in gastrocnemius muscles. Our findings showing that acidic preconditioning increased the release of IL-4, TGFβ1, and IL-10 led us to speculate that it might be related to a paracrine action of these progenitors which, once engrafted, increase the local levels of anti-inflammatory cytokines. In this sense, it has been reported that endothelial progenitor cells improved the neurological function of rats with intracerebral hemorrhage by decreasing pro-inflammatory cytokines such as IFN-γ, IL-6, and TNFα, and increasing anti-inflammatory cytokines such as TGFβ1 and IL-10 [31]. Moreover, Moubarik et al. suggested that endothelial progenitor cell-mediated attenuation of neurological injury after middle cerebral artery occlusion may include upregulation of neural protective cytokines, such as IGF-1, and downregulation of proBDNF, which is a proinflammatory factor [32].

Altogether, our findings show that acidic preconditioning improved ECFC survival and angiogenic activity in the presence of proinflammatory and damage signals highly present in the ischemic milieu and is an effective strategy to promote postischemia tissue regeneration in a murine model of type 2 diabetes.

## Conclusions

In the present study, we demonstrated that acidic preconditioning of human ECFC promoted adhesion and the release of proangiogenic molecules. In addition, this strategy attenuated the harmful effect induced by several molecules that are highly increased in the ischemic and inflammatory microenvironment, including MSU, extracellular histones, and TNFα, despite the different cell death programs triggered by each stimulus. Preconditioned ECFC exhibited an increased survival, in vitro expansion capacity, and angiogenic activity. This cytoprotective effect was also observed under high glucose conditions in vitro and, more interestingly, in a murine model of type 2 diabetes, where preconditioned ECFC were more efficient than untreated cells in restoring hind limb revascularization and reducing ischemia-related tissue damage and inflammation.

## Additional files

Additional file 1: ECFC cell death induced by histones or MSU is not affected under high glucose conditions. Nonpreconditioned ECFC (npECFC) were incubated with histones (A) or MSU crystals (B) at the indicated concentrations, in the presence or absence of high glucose, and cell death was analyzed after 24 h (*n* = 3–5). (DOCX 135 kb)

Additional file 2: ECFC adhesion under proinflammatory and high glucose conditions. Nonpreconditioned or preconditioned ECFC (npECFC or pECFC, respectively) were incubated with high glucose, TNFα, or their combination, and seeded onto fibronectin, collagen, or TNFα-activated HUVEC. After (A) 30 min or (B) 2 h, the number of adherent cells was counted. Results are expressed as percentage of nonpreconditioned ECFC (*n* = 5). *p < 0.05 vs untreated npECFC. ^#^*p* < 0.05 vs npECFC with the same treatment. (DOCX 478 kb)

Additional file 3: Histologic analysis of gastrocnemius muscles. PBS, nonpreconditioned, or preconditioned ECFC (npECFC or pECFC, respectively) were infused intravenously in normoglycemic and type 2 diabetic (T2D) mice 5 h after ischemia-inducing surgery. Histologic analysis of gastrocnemius muscles, stained with hematoxylin and eosin (H/E) or Masson’s trichrome, was performed after 14 days postischemia in normoglycemic and type 2 diabetic (T2D) mice (*n* = 6 per group). Original magnification, 100×. Scale bar = 20 μm. (DOCX 1573 kb)

## Abbreviations

bFGF: Basic fibroblast growth factor
CFSE: Carboxyfluorescein succinimidyl ester
DAMP: Damage-associated molecular patterns
ECFC: Endothelial colony-forming cells
EGF: Epidermal growth factor
EGM: Endothelial growth medium
FAK: Focal adhesion kinase
FITC: Fluorescein isothiocyanate
HFHS: High-fat high-sucrose
HUVEC: Human umbilical vein endothelial cells
IL: Interleukin
IOD: Integrated optical density
MSU: Monosodium urate
PBS: Phosphate-buffered saline
PDGF: Platelet-derived growth factor
PI: Propidium iodide
ROS: Reactive oxygen species
SDF: Stromal cell-derived factor
TGF: Transforming growth factor
TNF: Tumor necrosis factor
VEGF: Vascular endothelial growth factor
