# Supplementary material for: Acidic preconditioning of endothelial colony-forming cells (ECFC) promote vasculogenesis under proinflammatory and high glucose conditions in vitro and in vivo
Source: Stem Cell Res Ther. 2018 May 2;9:120. doi: 10.1186/s13287-018-0872-7 (PMC5930427; doi:10.1186/s13287-018-0872-7)
Supplement: Supplementary file 1 — ECFC cell death induced by histones or MSU is not affected under high glucose conditions. Nonpreconditioned ECFC (npECFC) were incubated with histones (A) or MSU crystals (B) at the indicated concentrations, in the presence or absence of high glucose, and cell death was analyzed after 24 h (n = 3–5). (DOCX 135 kb) [file 13287_2018_872_MOESM1_ESM.docx]

**Additional file 1: ECFC death induced by histones or MSU is not affected under high glucose conditions**


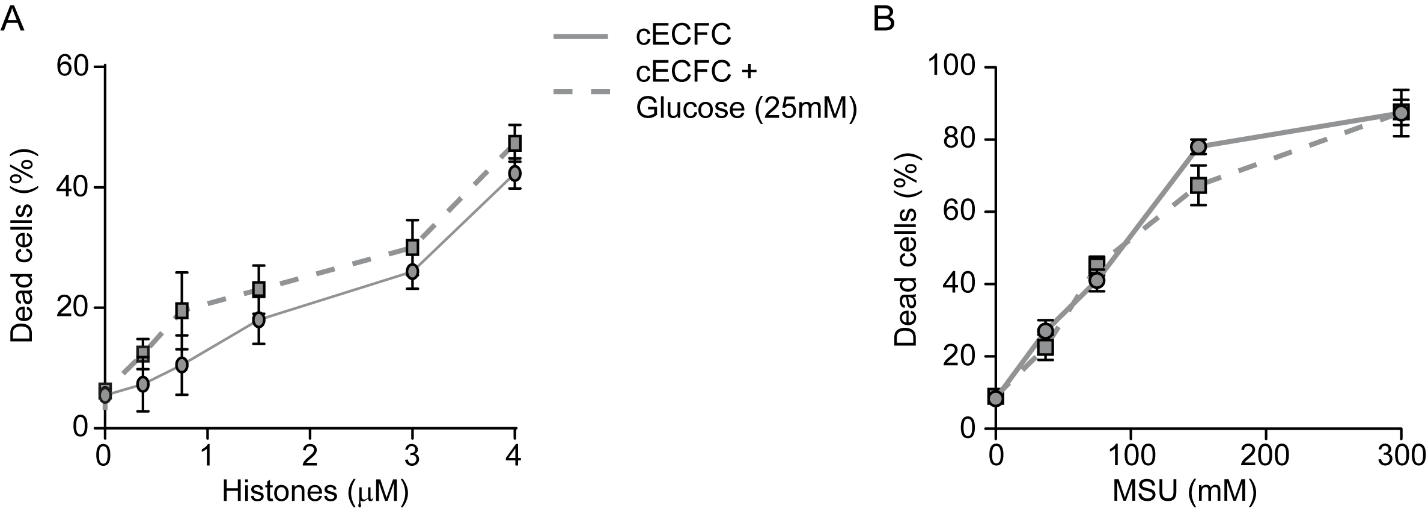


Non-preconditioned ECFC (npECFC) were incubated with histones (A) or MSU crystals (B) at the indicated concentrations, in presence or absence of high glucose and cell death was analyzed after 24 h (n=3-5).
