# Supplementary material for: Acidic preconditioning of endothelial colony-forming cells (ECFC) promote vasculogenesis under proinflammatory and high glucose conditions in vitro and in vivo
Source: Stem Cell Res Ther. 2018 May 2;9:120. doi: 10.1186/s13287-018-0872-7 (PMC5930427; doi:10.1186/s13287-018-0872-7)
Supplement: Supplementary file 2 — ECFC adhesion under proinflammatory and high glucose conditions. Nonpreconditioned or preconditioned ECFC (npECFC or pECFC, respectively) were incubated with high glucose, TNFα, or their combination, and seeded onto fibronectin, collagen, or TNFα-activated HUVEC. After (A) 30 min or (B) 2 h, the number of adherent cells was counted. Results are expressed as percentage of nonpreconditioned ECFC (n = 5). *p < 0.05 vs untreated npECFC. #p < 0.05 vs npECFC with the same treatment. (DOCX 478 kb) [file 13287_2018_872_MOESM2_ESM.docx]

**Additional file 2. ECFC adhesion under proinflammatory and high glucose conditions**

**
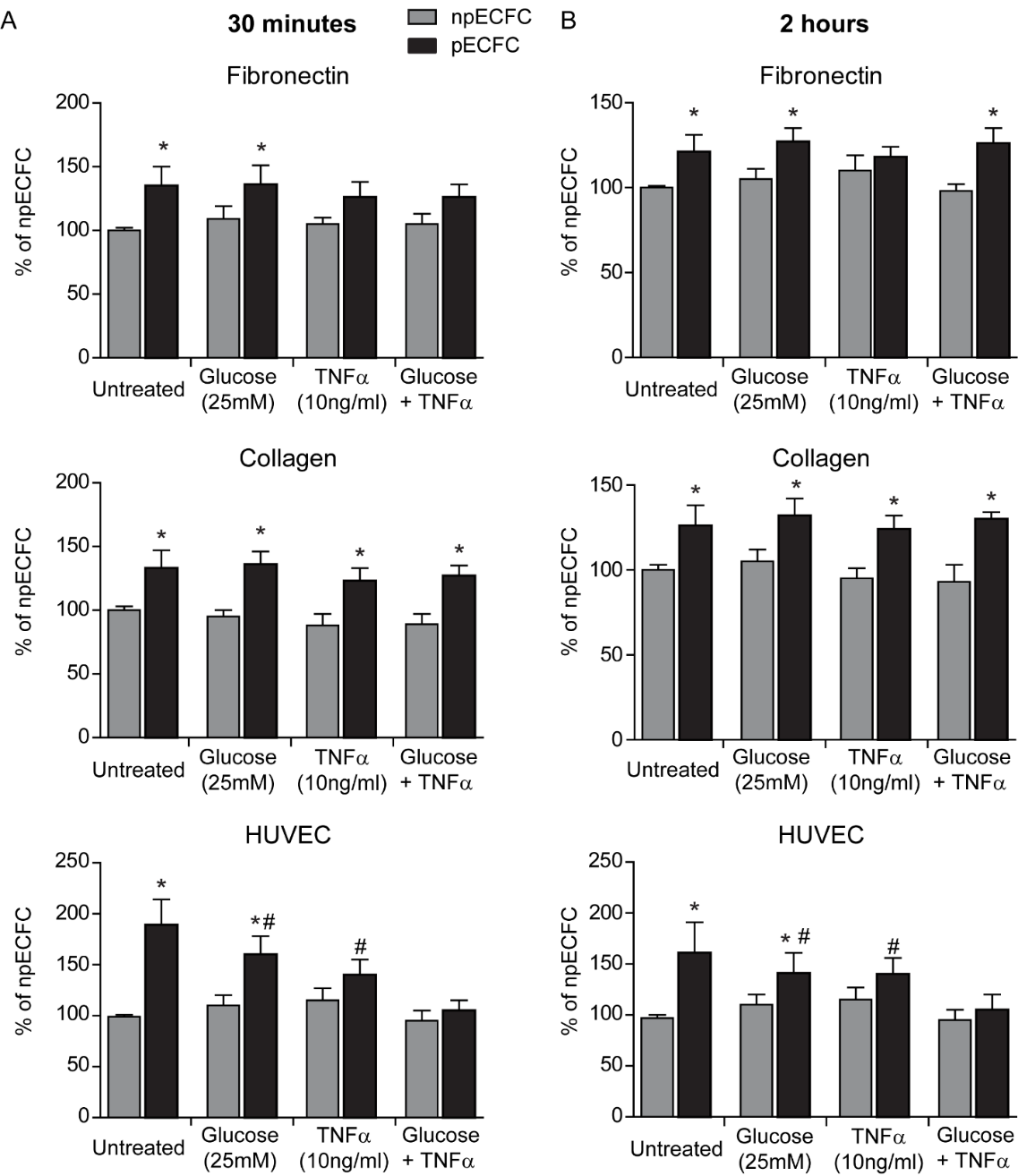
**

Non-preconditioned or preconditioned ECFC (npECFC or pECFC, respectively) were incubated with high glucose, TNFα or their combination and seeded onto fibronectin, collagen or TNFα-activated HUVEC. After (A) 30 minutes or (B) 2 hours, the number of adherent cells was counted. Results are expressed as percentage of non-preconditioned ECFC (n=5). *p<0.05 vs untreated npECFC. #p<0.05 vs npECFC with same treatment.
