# Supplementary material for: Acidic preconditioning of endothelial colony-forming cells (ECFC) promote vasculogenesis under proinflammatory and high glucose conditions in vitro and in vivo
Source: Stem Cell Res Ther. 2018 May 2;9:120. doi: 10.1186/s13287-018-0872-7 (PMC5930427; doi:10.1186/s13287-018-0872-7)
Supplement: Supplementary file 3 — Histologic analysis of gastrocnemius muscles. PBS, nonpreconditioned, or preconditioned ECFC (npECFC or pECFC, respectively) were infused intravenously in normoglycemic and type 2 diabetic (T2D) mice 5 h after ischemia-inducing surgery. Histologic analysis of gastrocnemius muscles, stained with hematoxylin and eosin (H/E) or Masson’s trichrome, was performed after 14 days postischemia in normoglycemic and type 2 diabetic (T2D) mice (n = 6 per group). Original magnification, 100×. Scale bar = 20 μm. (DOCX 1573 kb) [file 13287_2018_872_MOESM3_ESM.docx]

**Additional file 3. Histologic analysis of gastrocnemius muscles**


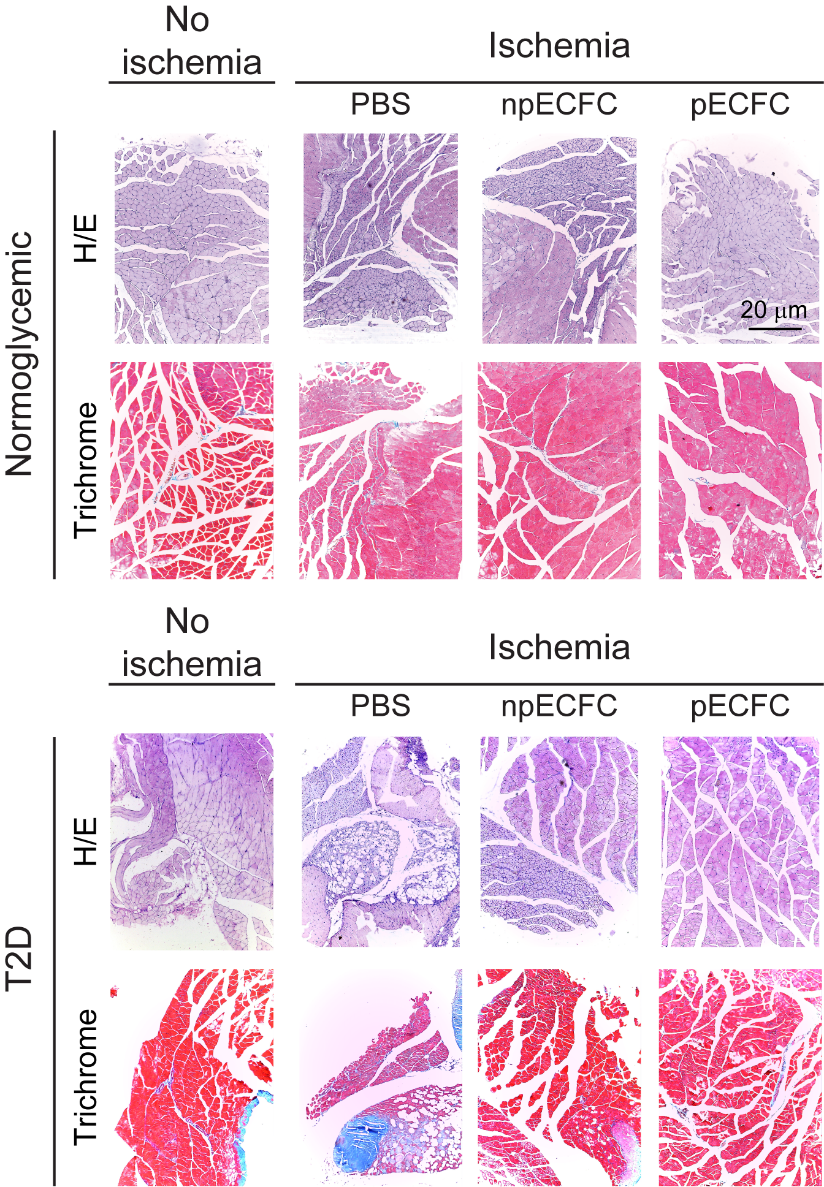


PBS, non-preconditioned or preconditioned ECFC (npECFC or pECFC, respectively) were infused intravenously in normoglycemic and type 2 diabetic (T2D) mice 5 h after ischemia-inducing surgery. Histologic analysis of gastrocnemius muscles, stained with hematoxylin and eosin (H/E) or Masson´s trichrome, was performed after 14 days post ischemia in normoglycemic and type 2 diabetic (T2D) mice (n=6 per group). Original magnification, 100x. Scale bar, 20µm.
